# Green Synthesis of Selenium, Zinc Oxide, and Strontium Nanoparticles and Their Antioxidant Activity - A Comparative In Vitro Study

**DOI:** 10.7759/cureus.50861

**Published:** 2023-12-20

**Authors:** Rajeshkumar Shanmugam, Jayasree Anandan, Ashwin K Balasubramanian, Rupa D Raja, Srivarsha Ranjeet, Pavithra Deenadayalan

**Affiliations:** 1 Department of Pharmacology, Saveetha Dental College and Hospital, Saveetha Institute of Medical and Technical Sciences, Saveetha University, Chennai, IND; 2 Department of Pharmacology, Saveetha Dental College and Hospitals, Saveetha Institute of Medical and Technical Sciences, Saveetha University, Chennai, IND

**Keywords:** zinc oxide nanoparticles (zno), green synthesis, antioxidant therapy, strontium nanoparticles, selenium nanoparticles

## Abstract

Background

Antioxidants are vital in reducing oxidative stress, a key factor in the pathogenesis of many chronic diseases, including cancer, cardiovascular disease, and neurodegenerative disorders. The aim of our study is to analyze and compare the oxidative potential of biosynthesized selenium, strontium, and zinc oxide nanoparticles (NPs).

Materials and methods

Selenium nanoparticles (SeNPs) were synthesized using 20 mM of sodium selenite as the precursor and 1 g each of *Cymbopogon citratus* and *Syzygium aromaticum* as reducing and stabilizing agents. Strontium nanoparticles (SrNPs) were synthesized with 30 mM of strontium chloride as the precursor and 1 g of *Acacia nilotica* as a reducing and stabilizing agent. Zinc oxide nanoparticles (ZnONPs) were synthesized using 30 mM of zinc nitrate as the precursor and 1 g each of *Cuminum cyminum* and *Syzygium aromaticum* as reducing and stabilizing agents. Selenium, strontium, and zinc oxide nanoparticles were characterized using Fourier-transform infrared spectroscopy (FT-IR) analysis. The antioxidant activity of biogenically synthesized strontium, selenium and zinc oxide nanoparticles was examined using the 2,2-diphenyl-1-picrylhydrazyl (DPPH) radical scavenging assay (DPPH assay) and hydroxyl radical scavenging assay (H_2_O_2 _assay).

Results

The FT-IR spectra of selenium nanoparticles revealed a peak at 3327.990 cm^−1^, strontium nanoparticles at 3332.331 cm^−1^, and zinc oxide nanoparticles at 3216.346 cm^−1^. The significant results for the green-synthesized selenium, strontium, and zinc oxide nanoparticles were observed in antioxidant assays. The results from the DPPH assay show that at the highest concentration of 50 µL, SrNPs exhibited 90.12 % inhibition, SeNPs displayed 90.12% inhibition, and ZnONPs showed 89.55% inhibition. In the H_2_O_2_ assay, at the highest concentration of 50 µL, SrNPs showed 87.43% inhibition, SeNPs displayed 85.11% inhibition, and ZnONPs exhibited 84.66% inhibition. SrNPs demonstrated a higher percentage of inhibition in both the DPPH and H_2_O_2_ assays. Maximum inhibitory activity was observed at the highest concentration. However, the prepared nanoparticles showed a slightly lower percentage of inhibition when compared to the standard.

Conclusion

Strontium nanoparticles synthesized based on *Acacia nilotica* demonstrated excellent antioxidant activity compared to the synthesized selenium and zinc oxide nanoparticles. Therefore, the study suggests that the produced strontium nanoparticles can serve as an antioxidant agent, owing to their remarkable free radical scavenging activity.

## Introduction

Nanotechnology has rapidly evolved, finding applications across diverse fields such as biology, chemistry, physics, and bioengineering [1]. Nanoparticles, produced through various methods, have gained attention for their unique properties [2]. Among them, metal nanoparticles, like selenium nanoparticles (SeNPs), strontium nanoparticles (SrNPs), and zinc oxide nanoparticles (ZnONPs), have shown promise in biomedical applications due to their antioxidant capabilities and ability to transition between oxidation states [3]. In nanoparticle synthesis, the biological approach stands out for its environmental friendliness and cost-effectiveness due to the utilization of microorganisms or medicinal plants, especially those with therapeutic properties, which have become preferred. Plant phytocompounds, known for their antioxidant and antibacterial properties, can be integrated into nanoparticles during production [4].

Selenium, a trace element in the human body, exhibits antioxidant, anti-inflammatory, and anti-diabetic properties. Selenium nanoparticles have been synthesized using medicinal plants to enhance their biomedical applications [5]. Similarly, strontium nanoparticles, known for applications in various fields, are synthesized using medicinal plants, offering benefits in cancer therapy and drug delivery [6]. Zinc oxide nanoparticles are versatile inorganic compounds with antibacterial properties [7]. Their safety in biological systems and selective targeting capabilities make them valuable in antimicrobial, antiviral, biomedical, and environmental applications [8].

The link between natural antioxidant consumption and a lower risk of diseases like cancer and heart disease has fuelled increased research into the antioxidant content of medicinal plants, fruits, and vegetables [9]. Lemongrass (*Cymbopogon citratus*, *Myrtaceae* family), clove (*Syzygium aromaticum L.*, *Gramineae* family), Arabic gum (*Acacia nilotica,* *Fabaceae* family), and cumin (*Cuminum cyminum*, *Parsley* family) are the focal points of this study. The flavonoid compound quercetin, present in *Cymbopogon citratus (C. citratus),* is highly known for its therapeutic properties, like antioxidant and anti-inflammatory properties [10]. *Syzygium aromaticum​​​​​​​*(*S. aromaticum)*, renowned for its phenolic compounds with antioxidant properties, plays a crucial role in enhancing the antioxidant potential for synthesizing nanoparticles [11]. *Acacia nilotica​​​​​​​ (A. nilotica)*, valued for its therapeutic properties, adds further diversity to the range of plant materials explored in this research [12]. The *Cuminum cyminum​​​​​​​ (C. cyminum)* herb belongs to the *Parsley *family; it has a traditional reputation as a digestive aid. Additionally, it exhibits potent antimicrobial, anti-diabetic, and antioxidant activities [13].

By combining green synthesis with the antioxidant properties inherent in medicinal plants, this study aims to unravel the combination that can be harnessed for potential applications in health and related fields. The insights gained from evaluating and comparing the antioxidant activity of SeNPs, SrNPs, and ZnONPs mediated by specific medicinal plants contribute to our understanding of the intricate interaction between nanotechnology and natural sources. These findings hold promise for developing pioneering alternatives to antioxidants with broader implications for sustainable and eco-friendly biomedical applications.

The primary objective of this research is to evaluate and compare the antioxidant activity of selenium nanoparticles mediated by* C. citratus *and* S. aromaticum*, strontium nanoparticles prepared using *A. nilotica*, and zinc oxide nanoparticles prepared using *C. cyminum*. This study aims to deepen our understanding of their antioxidant potential by exploring the symbiotic relationship between medicinal plants and nanoparticles. The insights gained may have implications for applications in health and related fields, emphasizing nanotechnology's role in harnessing medicinal plants' therapeutic properties.

## Materials and methods

Green synthesis of selenium nanoparticles

*C. citratus *leaf and *S. aromaticum* bud powder were purchased from the local supermarket in Poonamallee, Chennai, India. Subsequently, 1 g each of *C. citratus *and *S. aromaticum *was measured and added to 100 mL of distilled water. The mixture was boiled for 15 minutes using a heating mantle at 50 degrees Celsius and then filtered using Whatman No. 1 filter paper (Whatman plc, Maidstone, United Kingdom). Later, 20 mM (millimolar) of sodium selenite was measured and mixed with 50 mL of distilled water. To this, 50 mL of the filtered *C. citratus* and *S. aromaticum *herbal formulations were added. The mixture was then placed on a magnetic stirrer and stirred continuously for 48 hours at a rate of 600 rotations per minute (RPM). After 48 hours, centrifugation was performed for 10 minutes at 8000 RPM. The pellet containing selenium nanoparticles was then placed in a hot air oven to dry into a powder. Lastly, 100 milligrams of powdered nanoparticles were added to 10 milliliters of distilled water for further use.

Green synthesis of strontium nanoparticles

*Acacia nilotica* leaves were purchased from a supermarket in Poonamallee. Subsequently, 1 g of *A. nilotica* powder was combined with 100 mL of distilled water and subjected to boiling using a heating mantle at 50 degrees Celsius for 20 minutes. The resulting mixture was then filtered using Whatman filter paper. In a separate solution, 20 mM of strontium chloride was dissolved in 50 mL of distilled water. Subsequently, 50 mL of the filtered *A. nilotica *extract was added to the strontium chloride solution. The combined solution was placed on a magnetic stirrer and stirred continuously for 48 hours at 600 RPM. After the stirring period, centrifugation was conducted at 8000 RPM for 10 minutes. The pellet obtained after centrifugation was collected, and the supernatant was discarded. The pellet was then dried in a hot air oven to convert it into a powdered form. Finally, 100 mg of the powdered nanoparticle was mixed with 10 milliliters of distilled water for subsequent use. 

Green synthesis of zinc oxide nanoparticles

*C. cyminum* seed and *S. aromaticum *bud powder were purchased from a local supermarket in Poonamallee. Subsequently, 1 g each of *C. cyminum *and *S. aromaticum* were mixed with 100 mL of distilled water and boiled using a heating mantle at 50 degrees Celsius for 20 minutes. The resulting formulation was filtered using Whatman No. 1 filter paper. In a separate solution, 30 mM of zinc nitrate was prepared by mixing it with 50 mL of distilled water. This solution was then combined with 50 mL of the filtered *C.* *cyminum* and *S. aromaticum *formulation. The mixture was placed on a magnetic stirrer and continuously stirred for 48 hours at a rate of 600 RPM. Following this, a centrifugation process was carried out at 8000 RPM for 10 minutes. The pellet containing zinc oxide nanoparticles was collected and subjected to drying in a hot air oven to obtain it in a powdered form. After powdering, 100 mg of the nanoparticle powder was mixed with 10 mL of distilled water for later use.

Characterization 

The green synthesized selenium, strontium, and zinc oxide nanoparticles were characterized using a Fourier-transform infrared spectrophotometer (FTIR; Alpha II; Bruker, Billerica, Massachusetts) to identify the functional groups present in the synthesized nanoparticle solutions.

Antioxidant activity

*2,2-Diphenyl-1-Picryl Hydrazyl (DPPH*​​​​​​*) Radical Scavenging Assay (DPPH Assay)*

The DPPH assay was conducted using a methodology outlined in a previous study. We investigated the antioxidant activity of the synthesized zinc oxide, strontium, and selenium nanoparticles [14]. The DPPH assay was used for this evaluation.

Hydroxyl Radical Scavenging Assay (H_2_O_2_ Assay)

The assay was conducted following a methodology outlined in a prior study by Ganapathy et al. to investigate the antioxidant activity of the prepared selenium, strontium, and zinc oxide nanoparticles, utilizing a hydroxyl radical scavenging assay [15].

## Results

Green synthesized selenium, strontium, and zinc oxide nanoparticles

**Figure 1:**
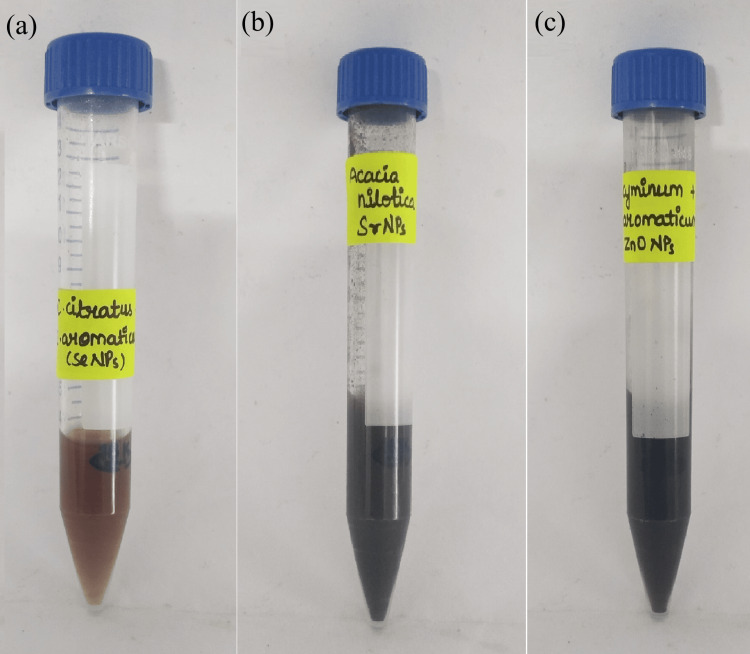
The image represents the collected pellet of (a) SeNPs from Cymbopogon citratu and Syzygium aromaticum, (b) SrNPs from Acacia nilotica, and (c) ZnONPs from Cuminum cyminum and Syzygium aromaticum SeNPs - selenium nanoparticles; SrNPs - strontium nanoparticles; ZnONPs - zinc oxide nanoparticles

After the synthesis of selenium nanoparticles from *C. citratus* and *S. aromaticum* (Figure 1 (a)), strontium nanoparticles from *A. nilotica *(Figure 1 (b)), and zinc oxide nanoparticles from *C. cyminum* and *S. aromaticum* (Figure 1 (c)), the nanoparticle solutions were subjected to centrifugation. After centrifugation, the collected pellet containing nanoparticles was stored for further use.

Fourier-transform infrared spectroscopy (FTIR) analysis of selenium, strontium and zinc oxide nanoparticles

**Figure 2:**
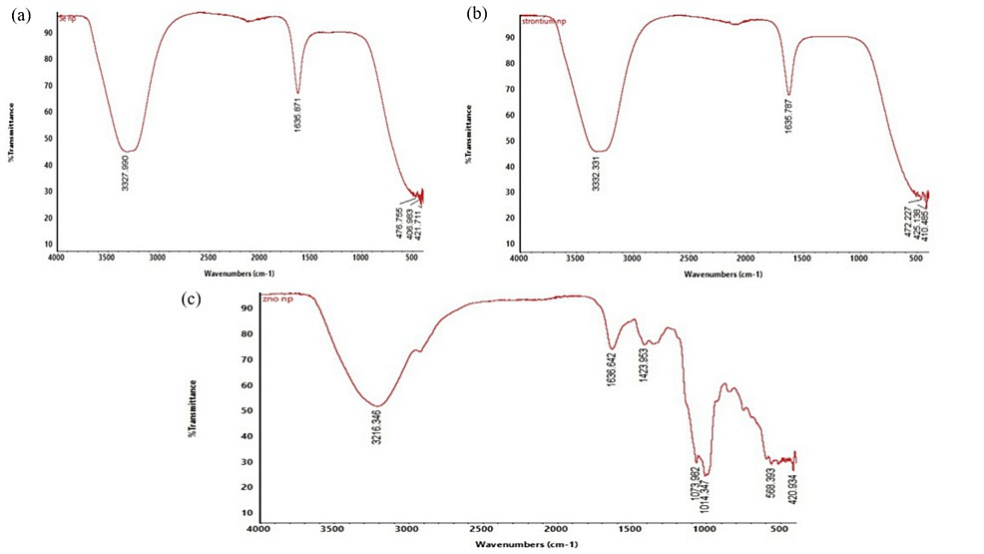
The FTIR analysis of (a) SeNPs, (b) SrNPs and (c) ZnONPs FTIR - Fourier transform infrared; SeNPs - selenium nanoparticles; SrNPs - strontium nanoparticles; ZnONPs - zinc oxide nanoparticles

An FTIR analysis was performed for synthesized selenium nanoparticles from *Cymbopogon citratus* and *Syzygium aromaticum*, strontium nanoparticles synthesized using* Acacia nilotica,* and zinc oxide nanoparticles prepared using *Cuminum cyminum* and *Syzygium aromaticum*. The FTIR spectra revealed diverse peaks at different wavenumbers in selenium nanoparticles (Figure 2a). The notable peaks were observed at 3327.990 cm^−1^, 1635.871 cm^−1^, 476.755 cm^−1^, 406.983 cm^−1^, and 421.711 cm^−1^. The broad peak observed at 3327.990 cm^−1^ was identified as a normal polymeric OH stretch of alcohol and hydroxy compound. The shorter peak at 476.755 cm^−1^ indicates the presence of S- S stretch polysulphide thiols and thio substituted compounds. The other peaks were observed as 406.983 cm^−1^ and 421.711cm^−1^, which describe various functional groups present in the plant extract.

The FTIR spectra of strontium nanoparticles exhibited different peaks at various wavenumbers (Figure 2b). The significant peaks were observed at 3332.331 cm^−1^, 1635.787 cm^−1^, 472.227 cm^−1^, 425.138 cm^−1^ and 410.485 cm^−1^. The wider peak observed at 3332.331 cm^−1^ revealed the presence of alcohol and hydroxy compounds of normal polymeric OH stretch. The peak at 1635.787 cm^−1^ represents the primary amine of NH bend in primary amino ether and oxy compound, and the peak at 472.227 cm^−1 ^detected the S-S stretch of polysulphides thiols and thio-substituted compounds. The varied peaks at 425.138 cm^−1^ and 410.485 cm^−1^ also revealed the presence of other phenolic compounds in the *Acacia nilotica-*mediated strontium nanoparticles.

In zinc oxide nanoparticles, the FTIR spectra showed various peaks at different wavenumbers (Figure 2 c). The peaks were observed at 3216.346 cm−1, 1636.642 cm^−1^, 1636.642 cm^−1^, 1423.953 cm^−1^, 1073.982 cm^−1^, 1014.374 cm^−1^ , 568.393 cm^−1 ^and 420.934 cm^−1^.The broader peak at 3216.346 cm^−1^ showed the presence of ammonium ion, which is a common inorganic ion, and 1636.642 cm^−1^ indicated the presence of organic nitrates in nitrogen oxy compounds in the simple hetero-oxy compounds group. The other peak at 1423.953 cm^−1^ is carbonate ion in common inorganic ions. Similarly, the peak at 1073.982 cm^−1^ is a C-O stretch, large rings of cyclic ether in ether and oxy compound. The peaks at 1014.374 cm^−1 ^and 568.393 cm^−1^ represent the presence of phosphate ion, and C-I stretch aliphatic ion compounds in aliphatic organohalogen compounds. The shorter peak at 420.934 cm^−1^ represents another functional group in the* Cuminum cyminum* and *Syzygium aromaticum-*mediated zinc oxide nanoparticles.

Antioxidant activity of selenium nanoparticles

**Figure 3:**
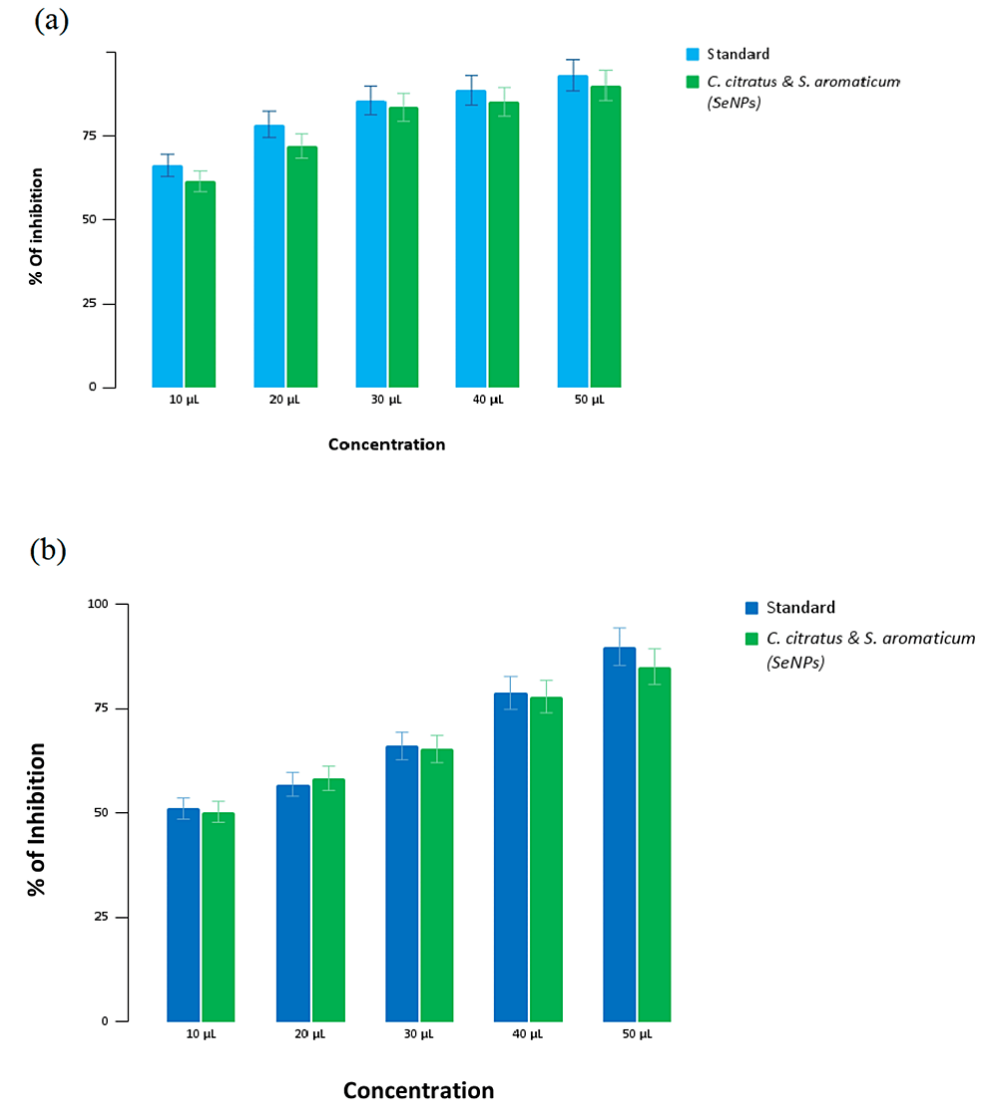
Antioxidant activity of selenium nanoparticles (a) DPPH assay, and (b) H2O2 assay DPPH - 2,2-diphenyl-1-picrylhydrazyl

In Figure 3a, the graph illustrates the antioxidant activity of the prepared selenium nanoparticles and a standard (ascorbic acid) against DPPH radicals in the DPPH assay. At the highest concentration of 50 µL (microlitres), the standard displays a 93.15% inhibition in radical activity, whereas the SeNPs show a slightly lower 90.12% inhibition. Conversely, at the lowest concentration of 10 µL, the standard exhibits a 66.25% inhibition, and the SeNPs show a slightly lower effect of 61.53% inhibition.

In Figure 3b, the bar graph depicts the antioxidant activity of the prepared SeNPs and the standard (ascorbic acid) against H_2_O_2_ radicals in the H_2_O_2_ assay. At the high concentration of 50 µL, the standard reveals an 89.9% inhibition rate, while the SeNPs show a slightly lower 85.1% inhibition. When the concentration is reduced to the lowest value of 10 µL, the standard's inhibition percentage is 51.1%, and the SeNPs display a 50.3% inhibition rate, which is very similar to that of the standard.

Antioxidant activity of strontium nanoparticles

**Figure 4:**
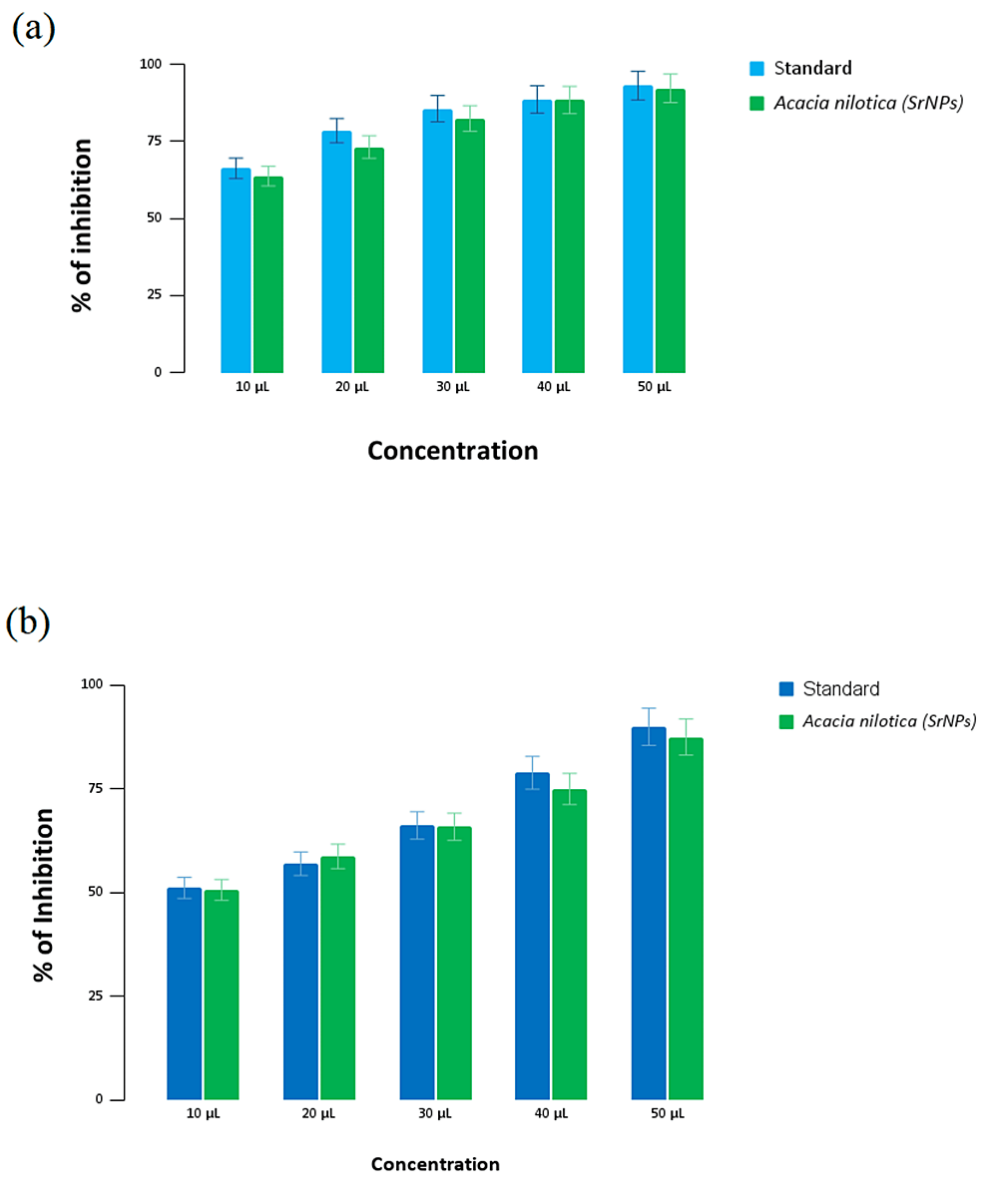
Antioxidant activity of strontium nanoparticles (a) DPPH assay, and (b) H2O2 assay DPPH - 2,2-diphenyl-1-picrylhydrazyl

Figure 4a shows the free radical scavenging activity of strontium nanoparticles and the standard (ascorbic acid) against DPPH radicals. At the highest concentration of 50 µL, the standard exhibits 92.26% inhibition, and the SrNPs show 90.12% inhibition. At the lowest concentration of 10 µL, the standard shows 66.25% inhibition, and SrNPs demonstrate 63.25% inhibition.

Figure 4b illustrates the antioxidant activity of the strontium nanoparticles and the standard (ascorbic acid) against hydrogen peroxide radicals in the H_2_O_2_ assay. At the maximum concentration of 50 µL, the standard has 89.9% inhibition, and the SrNPs showed 87.4% inhibition. At the minimum concentration of 10 µL, the standard shows 51.1% inhibition, and the SrNPs exhibit 50.6% inhibition.

Antioxidant activity of zinc oxide nanoparticles

**Figure 5:**
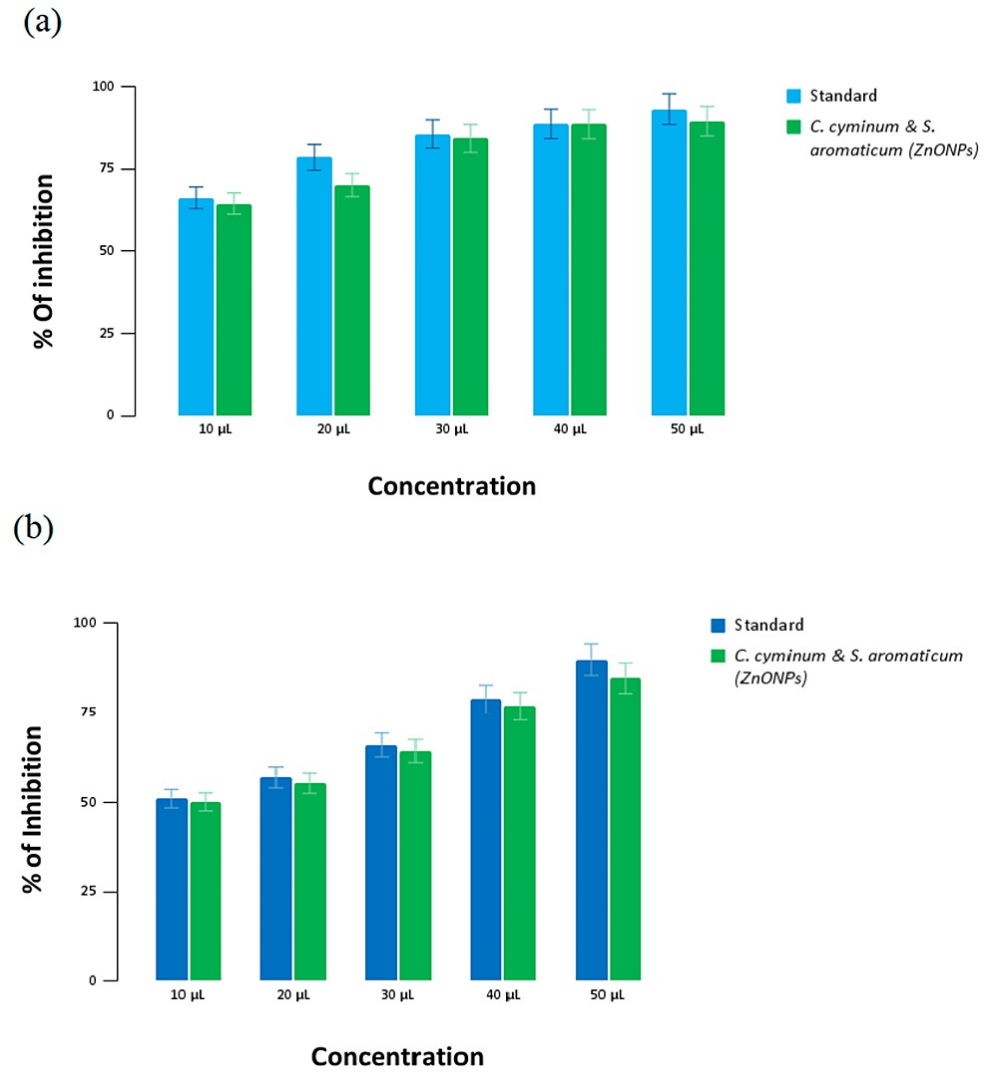
Antioxidant activity of zinc oxide nanoparticles (a) DPPH assay, and (b) H2O2 assay DPPH - 2,2-diphenyl-1-picrylhydrazyl

Figure 5a illustrates the antioxidant activity of the prepared zinc oxide nanoparticles and the standard (ascorbic acid) against DPPH radicals in the DPPH assay. At the highest concentration of 50 µL, the standard exhibits 93.15% inhibition, while ZnONPs show 89.55% inhibition. Conversely, at the lowest concentration of 10 µL, the standard displays 66.25% inhibition, and ZnONPs exhibit 63.25% inhibition.

Figure 5b depicts the antioxidant activity of the prepared zinc oxide nanoparticles and the standard (ascorbic acid) against H_2_O_2_ radicals in the H_2_O_2 _assay. At the highest concentration of 50 µL, the standard demonstrates 89.9% inhibition, whereas ZnONPs show 84.66% inhibition. At the lowest concentration of 10 µL, the standard exhibits 51.1% inhibition, and ZnONPs display 50.6% inhibition.

## Discussion

The current study demonstrated the antioxidant activity of SeNPs mediated by *Cymbopogon citratus* and *Syzygium aromaticum*, SrNPs mediated by *Acacia nilotica*, and ZnONPs mediated by *Cuminum cyminum* and *Syzygium aromaticum* formulation. SeNPs prepared using *C. citratus *and *S. aromaticum* formulation exhibited a 90.12% scavenging of free radicals at 50 µL concentrations in the DPPH assay and an 85.1% inhibition of free radicals at a 50 µL dose in the H_2_O_2_ assay. SrNPs synthesized using *A. nilotica* extract showed 90.12% inhibition of DPPH radical scavenging activity and 87.4% in the H_2_O_2 _assay at 50 µL concentrations. ZnONPs mediated by *C. cyminum* and *S. aromaticum* showed the percentage of inhibition in radical scavenging activity. At the highest concentration of 50 µL, it exhibited 89.55% in the DPPH assay and 84.66% in the H_2_O_2_ assay. In a comparative *in vitro *analysis of antioxidant activity using DPPH and H_2_O_2_ assays, the SrNPs mediated *Acacia nilotica* extract showed the maximum inhibition in free radical scavenging activity, which was slightly nearest to the standard (ascorbic acid) percentage. This may be due to the presence of phytochemical compounds present in *Acacia nilotica*, exhibiting strong antioxidant activity, whereas SeNPs and ZnONPs demonstrated somewhat lower activity. However, both selenium and strontium nanoparticles exhibited slightly lower antioxidant activity compared to the standard ascorbic acid.

In a prior study, SeNPs synthesized using *Theobroma cacao L.* bean shell extract demonstrated substantial antioxidant activity, as evidenced by their performance in both the 2,2’-azino-bis(3-ethylbenzothiozoline-6-sulphonic acid) (ABTS) assay and the ferric reducing antioxidant power (FRAP) assay. The presence of bioactive compounds in *Theobroma cacao L.* bean shell extract likely contributed to the robust antioxidant properties observed in SeNPs [16]. Selenium nanoparticles have shown significant promise as antioxidants, effectively scavenging free radicals and mitigating oxidative stress. This aligns with previous research emphasizing the influence of the synthesis method and source materials on the antioxidant efficacy of nanoparticles. Additionally, SeNPs functionalized with *Sargassum fusiforme* polysaccharides (SFPS) exhibited vigorous antioxidant activity in DPPH, H_2_O_2_, and ABTS assays [17]. The functionalization process not only contributed to the stability of SeNPs but also enhanced their antioxidant potential. The versatile antioxidant activity of SeNPs makes them appealing for various applications, including those involving reactive oxygen species (ROS) and oxidative stress. In the current study, SeNPs synthesized using* C. citratus *and *S. aromaticum* also demonstrated potent antioxidant activity, drawing the robustness of the green synthesis approach in harnessing the antioxidant potential of selenium nanoparticles.

Zinc oxide nanoparticles have gained attention not only for their well-established antibacterial properties but also for their intriguing antioxidant potential. In a previous study, ZnONPs biosynthesized from *Mangifera indica *(mango) leaves using an environmentally friendly approach displayed increased antioxidant effectiveness with rising concentrations [18]. The antioxidant capabilities of ZnONPs position them as versatile possibilities for applications in oxidative stress management. Furthermore, the study reported the antioxidant activity of ZnONPs mediated by *C. cyminum *and *S. aromaticum*. These nanoparticles exhibited significant antioxidant potential, aligning with the recognized role of zinc as an effective antioxidant agent. The antioxidant properties of ZnONPs are crucial not only for potential therapeutic applications but also for their utility in preventing oxidative damage in various biological systems.

Strontium nanoparticles have demonstrated remarkable antioxidant properties, as evidenced by their performance in free radical scavenging assays. In the comparative analysis conducted, SrNPs mediated by *Acacia nilotica *exhibited the highest free radical scavenging activity among the nanoparticles studied. This outcome emphasizes the potential of green-synthesized SrNPs for applications in antioxidant therapies. Moreover, in a separate study, strontium nanoparticles synthesized with Oolong tea exhibited substantial antioxidant activity in DPPH and H_2_O_2_ assays [19]. Similarly, SrNPs mediated by *A. nilotica *demonstrated remarkable and excellent antioxidant properties. The ability of SrNPs to quench free radicals and reduce oxidative stress markers positions them as promising evidence for diverse biomedical applications, ranging from cancer therapy to environmental sustainment.

In the final analysis, the demonstrated antioxidant potential of selenium nanoparticles, zinc oxide nanoparticles, and strontium nanoparticles in these studies have drawn the versatility and applicability of green-synthesized nanoparticles in effectively managing oxidative stress. These findings not only enhance our understanding of the therapeutic applications of nanoparticles but also pave the way for further research and development in the field of nanomedicine [20,21] and antioxidant [22] therapy. The environmentally friendly and cost-effective green synthesis approach [23], utilizing medicinal plants, adds a sustainable dimension to the production of these nanoparticles, making them promising evidence for various biomedical applications.

Limitations

In the present study, we conducted and compared in vitro analyses to assess the antioxidant activity of SeNPs, SrNPs, and ZnONPs using DPPH and H_2_O_2 _assays. To unravel the active ingredients and broaden our understanding, additional *in vitro* analyses, such as anti-inflammatory and antimicrobial properties, could be explored. Further comprehensive insights into the effects of these nanoparticles can be gained through subsequent *in vivo* investigations, including animal studies and clinical trials.

## Conclusions

In conclusion, the strontium nanoparticles synthesized using *A. nilotica* demonstrated significant antioxidant activity in both DPPH and hydrogen peroxide radical scavenging assays compared to the *C. cyminum *seeds and *S. aromaticum *mediated zinc oxide nanoparticles and selenium nanoparticles synthesized through *C. citratus* leaves and *S. aromaticum*. Among the three nanoparticles, zinc oxide nanoparticles showed comparatively less antioxidant activity in both DPPH and hydrogen peroxide radical scavenging assay. The antioxidant activity of the synthesized nanoparticles increased with an increase in nanoparticle concentration. This study suggests that green-synthesized strontium nanoparticles may offer a promising alternative in various therapeutic applications, presenting advantages over commercial synthetic drugs that often come with undesirable side effects.
